# Implantoplasty Positively Influences Biofilm Removal During Toothbrush: An In Vitro Study

**DOI:** 10.1111/idh.70063

**Published:** 2026-04-10

**Authors:** Robson Iralla Rezende, Cleverson O. Silva

**Affiliations:** ^1^ Universidade Estadual de Maringá Brazil

**Keywords:** dental devices, dental implants, dental plaque, home care, toothbrushing

## Abstract

**Purpose:**

To analyse the effect of implantoplasty on the efficacy of brushing dental implants.

**Methods:**

Acrylic hemi‐arches were made to simulate a horizontal bone loss condition in an implant in position 46, exposing 3 mm of its cervical intra‐osseous portion. Implantoplasty was performed on the implant surfaces of the test group, and no surface modification was performed on the control group. The exposed portion of both groups was coated with carbon spray to simulate dental biofilm. Each site was analysed before and after brushing for a carbon‐free area. Buccal/oral sites were brushed with toothbrushes while mesial/distal sites were brushed with interdental brushes. The analysed area consisted of a 6 mm^2^ rectangle divided into three thirds (cervical, middle and apical) on each of the four sites.

**Results:**

On buccal/oral sites of the test group (implantoplasty), brushing removed an average of 43.11% of the biofilm. In contrast, on the buccal/oral sites of the control group (no implantoplasty), brushing removed 36.09% (*p < 0.001*). On proximal sites of both test and control groups, brushing removed an average of 32.1% and 32.29% of the biofilm, respectively (*p = 0.728*).

**Conclusion:**

Implantoplasty positively influenced the removal of simulated biofilm on buccal/oral sites of dental implants.

## 1 Introduction

The rehabilitation of edentulous areas by prosthetics on implants has grown exponentially [1]. Data from 2018 show that almost 20 million implants are sold annually, with almost 200 million implants sold since 2003 to the present day [1]. However, peri‐implantitis (PI), a chronic inflammatory disease that affects peri‐implant tissues, has a high prevalence, ranging from 19% to 40% of patients, depending on the criteria applied [2, 3]. Many treatment modalities have been presented with the aim of reversing the inflammatory process and functionally preserving the implants affected by the disease [4, 5, 6].

Among treatment modalities, implantoplasty is a procedure with the objective of smoothening the implant surface through wear of its threads. In this way, the retentive areas that favour the accumulation of plaque are removed, facilitating mechanical cleaning by toothbrushes [7, 8]. It is known that implantoplasty reduces the implant diameter and results in different surface roughness, depending on the method applied [9]. However, implantoplasty doesn't seem to be associated with mechanical or biological complications, in addition to having a high success rate [6, 7, 8, 9, 10]. Also, a recent review reported a reduction in probing depth in sites treated with implantoplasty [11]. However, no study presented the impact of implantoplasty on the mechanical action of toothbrushes.

Although many studies have compared the effect of mechanical, chemical, and laser methods in the decontamination of dental implant surfaces, there is no consensus on a gold standard method [12, 13]. Even so, available research addresses mostly cleaning methods applied by professionals [11, 14, 15]. The literature is scarce of works that evaluate the effect of homeware brushing in the removal of biofilm on titanium. Park et al. [16] brushed surface‐treated titanium disks with adjunctive use of dentifrice. However, the titanium disks used in the research did not reproduce the limitations found in the macrostructure of dental implants. Elter et al. [17] brushed in vivo abutments, previously treated, in order to evaluate the removal of biofilm through toothbrushing. However, there was no standardization of the brushing method regarding the technique used, number of movements, and adjunct use of dentifrices. Batalha et al. [18] evaluated the effect of brushing dental implants in vitro with saline solution associated with professional decontamination means, such as abrasive jets and photodynamic therapy. However, there was no analysis of implants with surfaces modified by implantoplasty.

Finally, there are no studies that analyse whether implantoplasty facilitates brushing the surface of the dental implant when this is performed using toothbrushes and interdental brushes. That said, this research aims to analyse the influence of implantoplasty on the subsequent mechanical removal of simulated biofilm from dental implants in vitro.

## 2 Material and Methods

The tests were carried out on acrylic hemi‐arches (JET/Classico/Brazil) that simulated a condition of horizontal bone loss, similar to that caused by peri‐implantitis, in an implant in position 46, with neighbouring teeth presented. The simulated bone loss was 3 mm height, exposing the cervical 3 mm of the implant's intraosseous portion of the buccal, oral, mesial and distal sites. A cylindrical screw‐shaped, grade IV titanium implant with a sprayed etched surface (Strong SW, S.I.N., São Paulo/Brazil) was used, centrally positioned in the defect area. The implants' dimensions were 10/3.75/4.1 mm (length, diameter, external hexagonal connection). In the test group (*n* = 15), the implants were previously submitted to implantoplasty. In the control group (*n* = 15), implants did not receive any type of surface modification.

Implantoplasty was performed by a single experienced operator (RIR), outside the acrylic hemi‐arches, similarly to a previously published method [19]. Diamond burs (model 3118‐Microdont/Brazil) with decreasing grit sizes (107, 46, 25 μm) were used at 200.000 rpm, under abundant irrigation. After implantoplasty, the implant surface was not quantitatively analysed. The procedure for the first bur (107 μm) was done until the threads of the implant could not be identified by 4× visual magnification (magnifying glass Bio‐art/Brazil). There was no time limit for the use of each bur. For the consecutive burs (46, 25 μm), the procedure was done until a polished surface was achieved. Finally, the surface finishing was performed with an Arkansas stone (American Burs/Brazil) at 50.000 rpm, until a regular and polished surface was obtained. Each set of burs was used only once.

A single acrylic crown with a rotational abutment was made and positioned on the implant before initiating the tests. In order to reproduce this position on every implant, an addition silicone putty impression (Elite HD/Zhermack/Italy) was made and the buccal portion of the mould was removed, creating a template of the oral surfaces. In this way, it was possible to install the crown in the same position on every implant. The biofilm simulation was performed according to a previously published method [20]. A carbon spray layer (Artspray‐Bausch/Germany) was applied over the implant's surfaces, outside the acrylic hemi‐arch. After fixing the carbon, the four sites (buccal, oral, mesial and distal) were marked with a permanent marker pen (Pilot/Brazil) on the surface of the external connection of the implant. This mark didn't influence the area of interest, and aimed to guide the operator in repositioning the implants in the acrylic hemi‐arches. All sites were photographed before the marking. Each photograph was taken with a periodontal probe (North Carolina/Hu‐Friedy/United States) positioned beside the implant, to serve as a known measurement reference. Next, the implants were repositioned on the acrylic hemi‐arches, respecting the exposure of the 3 cervical mm and the direction of each site.

Implant brushing was performed with the hemi‐arches positioned on a table. A single experienced operator (RIR) performed the brushing. The buccal and oral sites were brushed with a conventional toothbrush (Curaprox 5460/Switzerland), without any force measurement, according to the modified Stillman technique, performing 10 strokes, five in each direction, mesio‐distal and cervico‐occlusal (back and forth) (Figure 1). The mesial and distal sites were brushed with 7.5 mm diameter cylindrical interdental brushes (Curaprox CPS 507/Switzerland), performing five horizontal strokes (back and forth) on mesial and distal sites, according to a previous publication [20] (Figure 1). Each set of brushes was used once per hemi‐arch. After brushing, the implants were removed from the acrylic hemi‐arch with the aid of an implant connection, which in turn was positioned in a digital screwdriver for connections previously fixed on a flat and rigid surface. Then, the implants were photographed on their respective sites (buccal, oral, mesial and distal). The photographs were taken, before and after brushing, with the following set: DSLR body (Canon/T3i), 100 mm/f2.8 macro lens (Canon), ring flash (Canon‐MR 14EX) with cross‐action polarizing filter (Cross‐Polar. Lithuania/UE). The set was stabilized on a tripod, and the lens was parallel to the implant at a fixed distance of 30 cm. In order to control the light conditions, the photographs were taken in a dark room, with the following configuration: aperture 16, speed 1/100, spot metering mode, white balance in flash mode and ISO 400. All samples, before and after brushing, for both groups (test/control), were edited following the same editing flowchart, as follows: scale adjustment, sample delimitation, contrast adjustment, colour threshold and selection of the area of interest.

**FIGURE 1:**
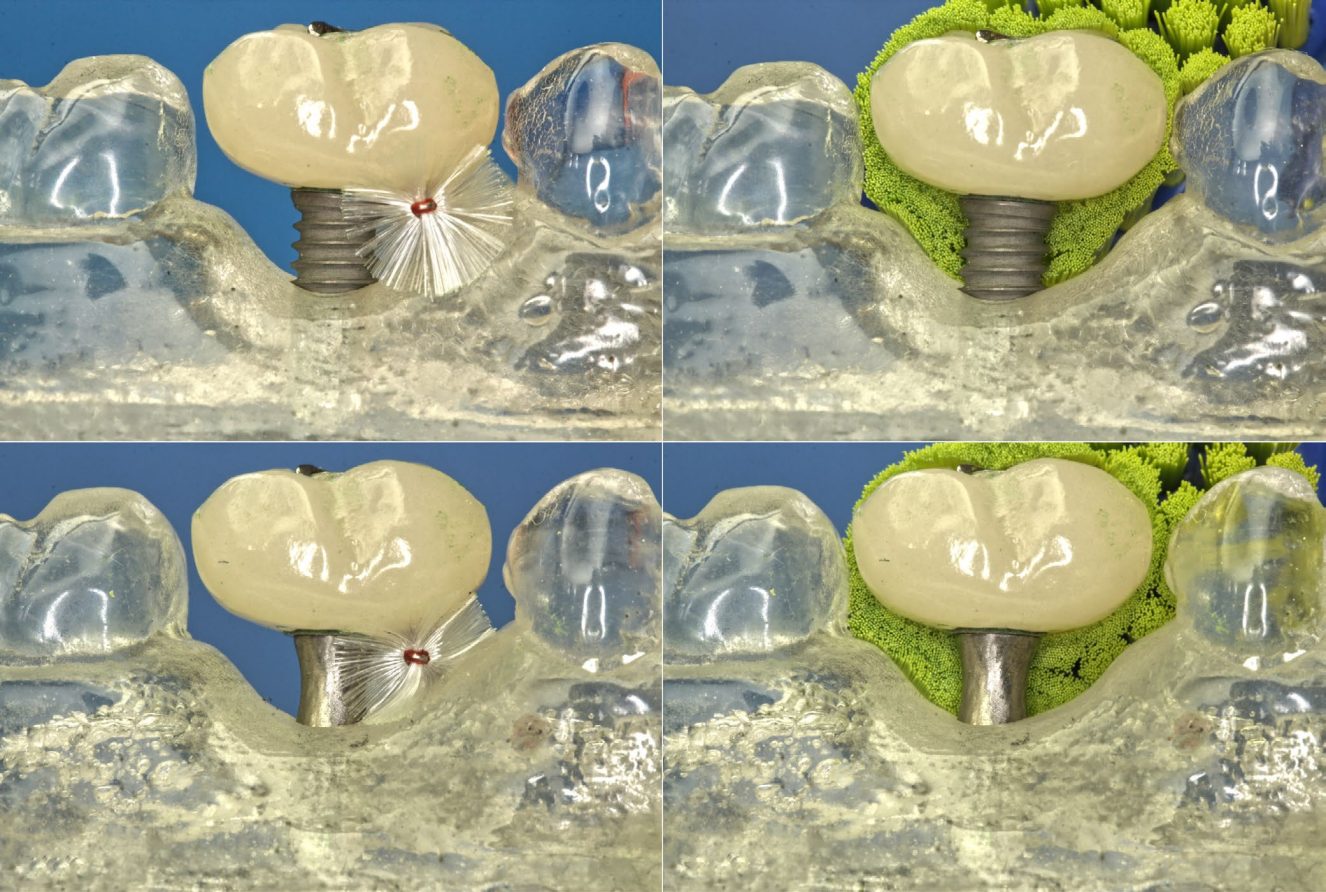
Proximal site brushing of test (lower left) and control groups (upper left). Oral site brushing of test (lower right) and control groups (upper right).

Altogether, 60 photographs of the test group and 60 of the control group were taken. The buccal and oral sites were grouped in the ‘buccal/oral sites’ group (*n* = 30), and the mesial and distal surfaces were grouped in the ‘proximal sites’ group (*n* = 30).

To quantify the brushing action, the method published by Tuna [20] (2019) was modified. An area of 3 × 2mm (6 mm [2]), from the most central and cervical point of the intraosseous portion of the implant, was cut from each photograph to serve as a sample of each site (Figure 2). For secondary analysis purposes, each sample was subdivided into three areas of 1 × 2mm (2 mm [2]), namely cervical, middle and apical.

**FIGURE 2:**
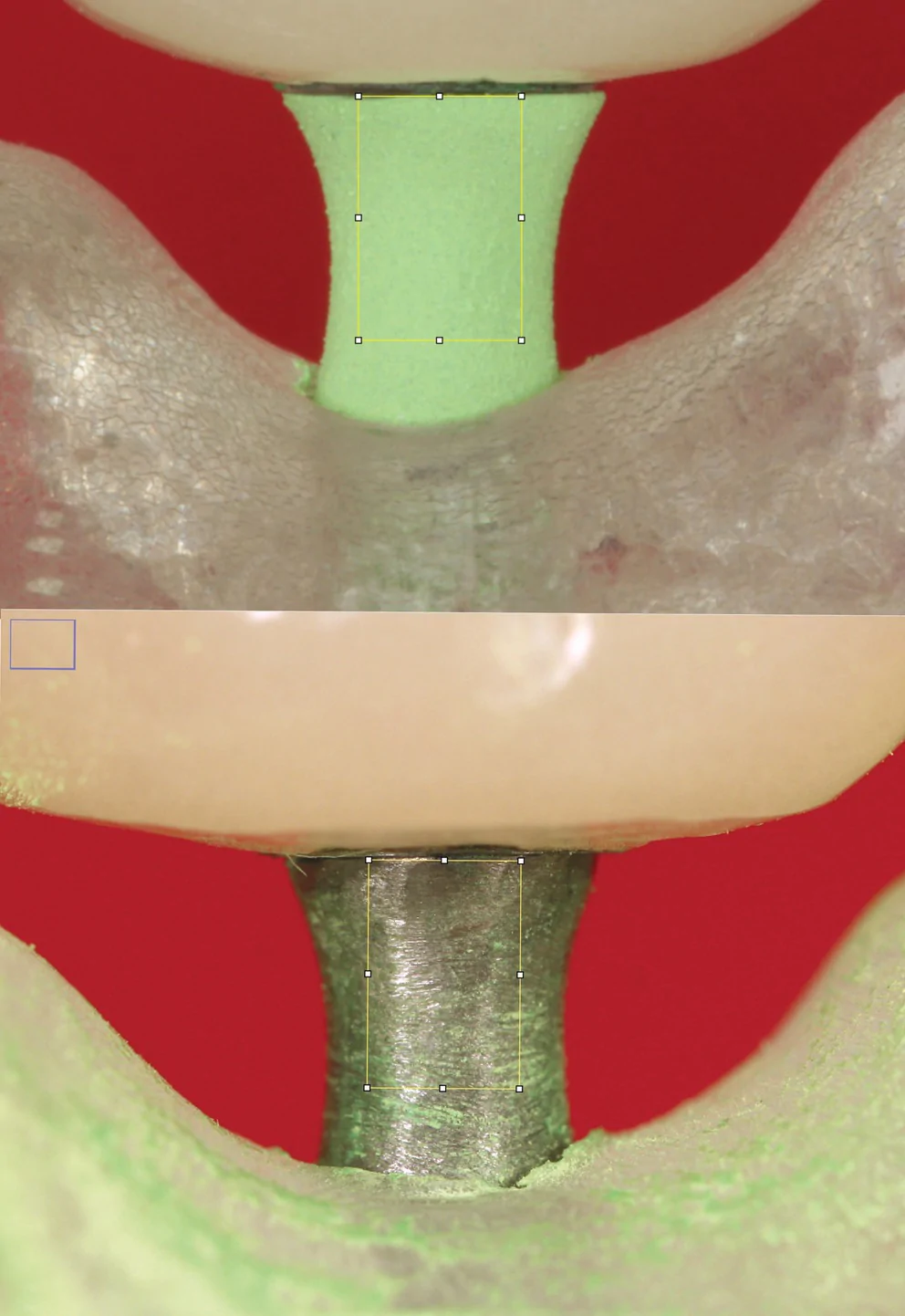
Area of interest selected for sample before (upper) and after (lower) brushing. Photograph without cross‐action polarizing filter.

The carbon green colour pixels contained in each sample were quantified before and after brushing. The difference in the percentage of pixels between the samples represented the brushing effect. To avoid selection bias, the green colour selection threshold was pre‐established according to an algorithm suggested in a previous publication (Figure 3) [21]. Editing and data collection were performed using the ImageJ software (version 1.53. National Institutes of Health/USA).

**FIGURE 3:**
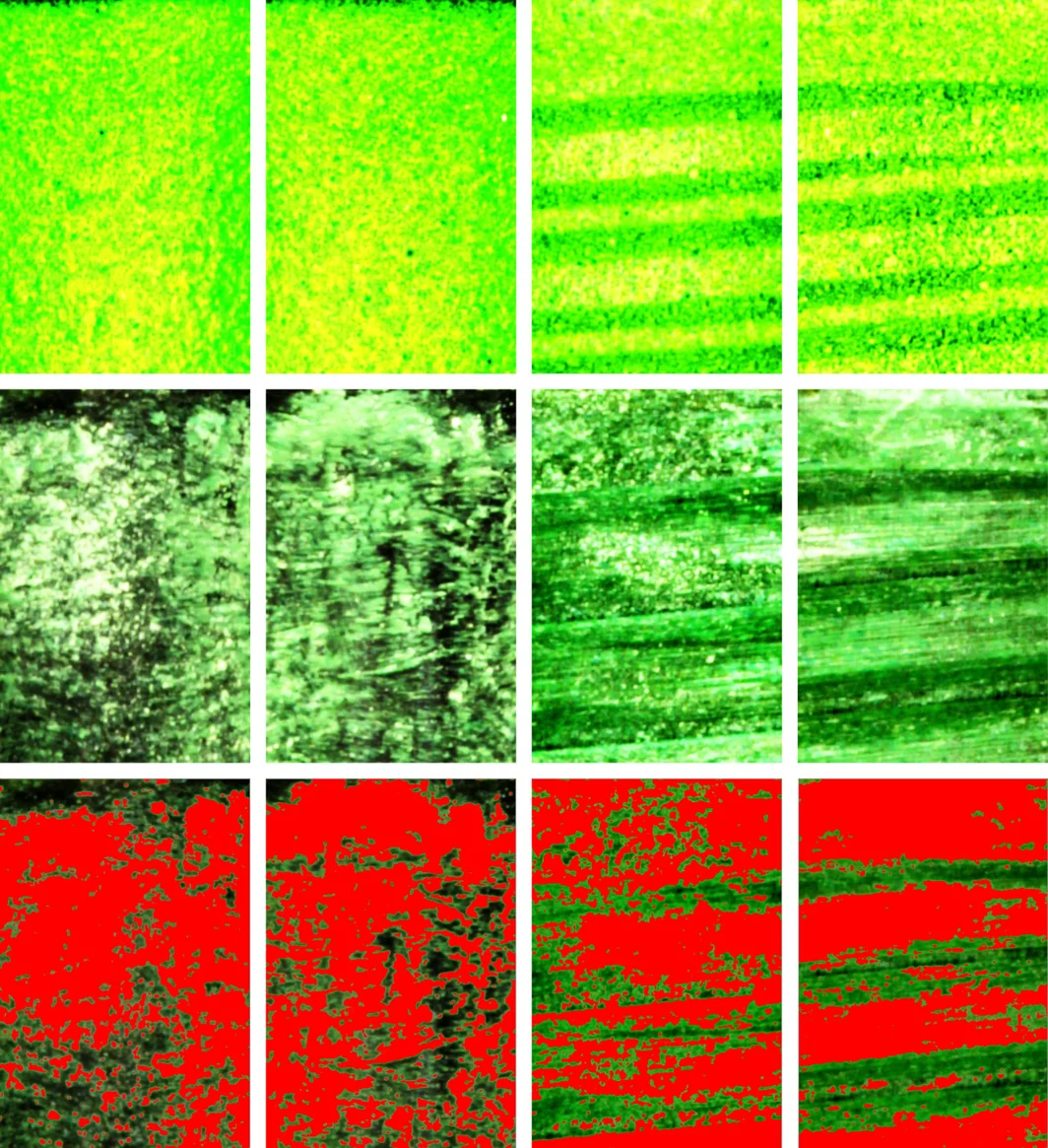
Upper row: Samples before brushing. Center row: Samples after brushing. Lower row: Carbon colour threshold selection (in red). First column: Oral/buccal surface of test group. Second column: Proximal surface of test group. Third column: Oral/buccal surface of control group. Fourth column: Proximal surface of control group.

A convenience sample of 30 implants was used in this research, with 15 implants allocated to each group (test and control). After comparison, a post hoc test was performed to obtain the power of the analysis.

Data were subjected to normality (Shapiro–Wilk) and homoscedasticity (Levene) tests, and presented as mean and standard deviation. Comparison of proximal sites between groups (test and control) was performed using Student's *t*‐test for independent groups. In contrast, the buccal/oral sites were compared using the Mann–Whitney test, due to the violation of normality/homoscedasticity assumptions. For further analysis, the implant's sites were divided into thirds (cervical, middle and apical). Comparisons between groups of proximal and buccal/oral sites, according to the thirds, were performed using factorial ANOVA with Bonferroni correction and Tukey's test. Intragroup comparison of thirds in the test group was performed using ANOVA for repeated measures, with Bonferroni correction and Tukey's test. On the other hand, in the control group, the Friedman test was used due to the violation of the normality/homoscedasticity assumptions. The significance used in the tests was 5% (*p < 0.05*), and all data were analysed using the statistical software Jamovi (version 1.6.23.0). The power analysis of the tests was performed using the G*Power 3.1 software.

## 3 Results

The percentage values of simulated biofilm removed in the test and control groups are presented in Tables 1 and 2. The *post hoc* analysis of the test power resulted in values ≥ 80%. Implantoplasty positively influenced the simulated biofilm removal on buccal/oral sites, but not on proximal sites. On average, 43.11% (SD±5.52) and 36.09% (SD±6.29) of the simulated biofilm was removed from the buccal/oral sites in the test and control groups, respectively. This difference was significant (Mann–Whitney; t = 20.50; df = 28; *p < 0.001*). On the other hand, there was a removal of 32.10% (SD±8.08) and 32.29% (SD±8.21) of the simulated biofilm from the proximal surfaces in the test and control groups, respectively. This difference was not significant (Student; t = 0.35; df = 28; *p = 0.728*) (Table 1).

**TABLE 1:** Percentage of simulated biofilm removal (mean ± SD) on buccal/oral and proximal sites in test and control groups.

Sites	Test	Control	*p*	Effect size (95% CI)
Buccal/oral	43.11 ± 5.52	36.09 ± 6.29	< 0.001^a^	2.86^c^ (−0.91, −0.62)
Proximal	32.10 ± 8.08	32.29 ± 8.21	0.728^b^	0.13^c^ (−0.59, 0.84)

^a^ Mann–Whitney test.

^b^ Student *t*‐test.

^c^ Cohen's d.

**TABLE 2:** Percentage of simulated biofilm removal (mean ± SD) in the test and control groups, on buccal/oral and proximal surfaces in relation to the thirds.

Sites	Thirds	Test	Control	*p*	Cohen's d (95% CI)
Buccal/oral	cervical	43.84 ± 7.25	35.5 ± 5.09	0.003	1.41 (0.65, 2.16)
	medium	41.4 ± 3.76	35.15 ± 5.75	0.075	1.05 (0.31, 1.80)
	apical	44.08 ± 4.93	37.61 ± 7.83	0.056	1.09 (0.34, 1.84)
Proximal	cervical	25.6 ± 7.48	27.56 ± 5.00	1.000	−0.28 (−1.01, 0.45)
	medium	34.23 ± 5.72	31.72 ± 8.44	0.922	0.36 (−0.37, 1.09)
	apical	36.49 ± 6.75	37.57 ± 7.82	0.998	−0.15 (−0.88, 0.57)

*Note:* ANOVA and Bonferroni correction.

When the sample was divided into thirds (Table 2), it was shown that implantoplasty significantly favoured simulated biofilm removal only in the cervical third of buccal/oral sites of the test group (between groups) (Student, t = 3.85, df = 85; *p = 0.003*). Intragroup analysis of cervical, middle and apical thirds of proximal sites showed that the cervical third presented the worst results, of both test (ANOVA, F = 11.61; df = 2; *p < 0.001*) and control groups (ANOVA, F = 12.51; df = 2; *p < 0.001*) (Figure 4). There was no statistical difference in relation to the thirds on the buccal/oral sites.

**FIGURE 4:**
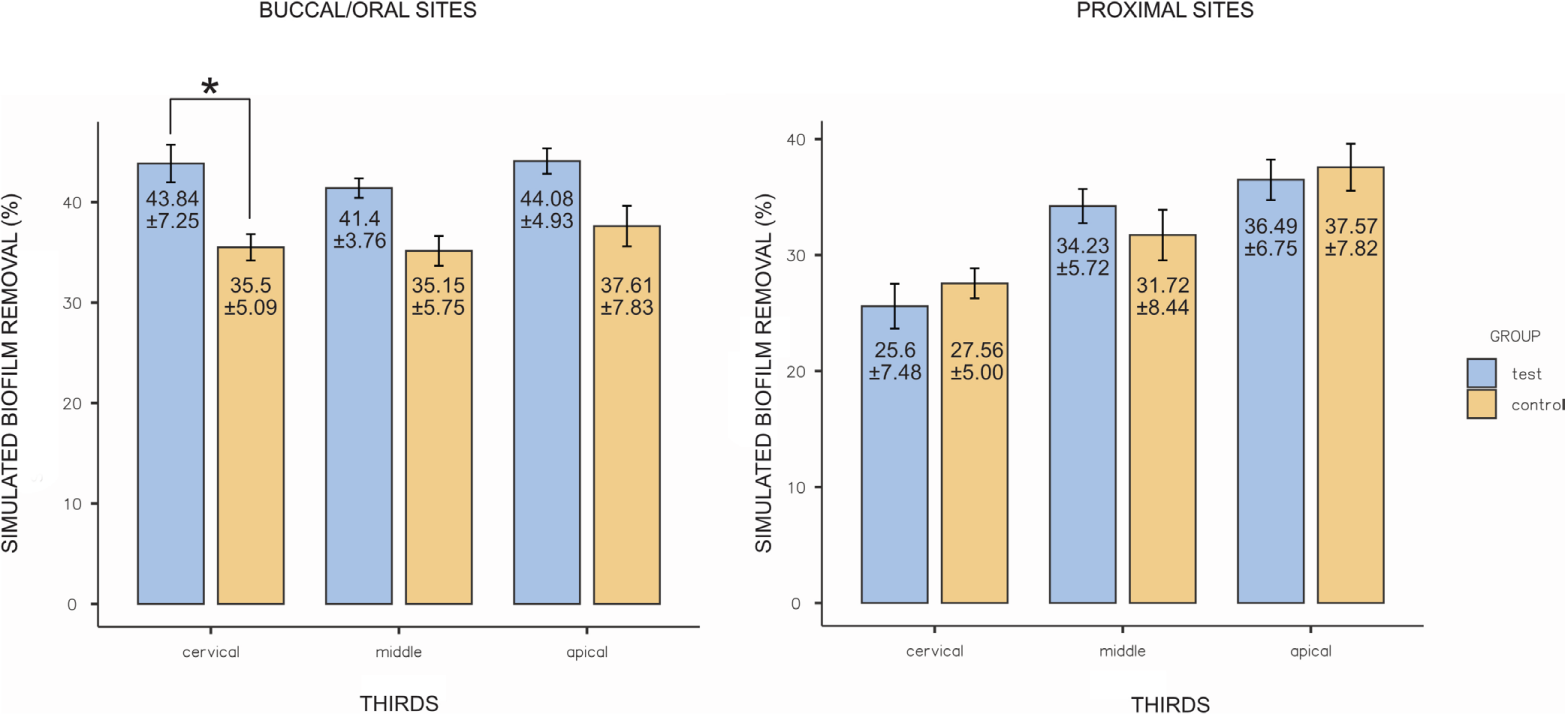
Intragroup analysis of simulated biofilm removal (mean/SD) in cervical, middle and apical thirds of the test and control groups. * *p* = 0,003.

## 4 Discussion

The present study evaluated the influence of implantoplasty on the removal of simulated biofilm on buccal/oral and proximal sites of dental implants. According to the authors' knowledge, the proposal to quantitatively evaluate the effect of simulated brushing on dental implants submitted to implantoplasty in vitro is unprecedented.

In vitro studies have an important role in dentistry, as they allow us to create and analyse situations that would be difficult to standardize in vivo. On the other hand, it is not possible to infer the results found from preclinical studies on clinical situations. Biofilm simulation using carbon spray proved to be an easy method to perform and effective in covering the implant surface of the test and control groups. Its colour contrast facilitated the identification of the cleaned areas after brushing. The use of carbon powder to simulate oral biofilm has already been reported in the literature on dental enamel, resin and ceramic surfaces [20, 22, 23, 24]. On the other hand, this method is different from in vivo biofilm with regard to its adherence to the titanium surface and its resistance to the mechanical action of brushing.

This study modified the method applied by Tuna et al. (2019) [20]. The photographs were taken with a cross‐action polarizing filter coupled to the flash (Cross‐Polar. Lithuania/EU). This measure aimed to reduce reflection areas, which would not be identified and measured. The images were methodically processed in order to eliminate selection biases of the area covered by spray. Finally, it was opted for an open domain software, already used in other studies, to process and quantify the areas of interest [17, 23].

According to Romeo et al. (2004), one of the purposes of implantoplasty is to facilitate the mechanical action of brushes [8]. This principle was confirmed in the present study on the buccal/oral sites (*p < 0.001*), but not on the proximal sites (*p = 0.728*). According to the results, on average, 43% of the buccal/oral sites were cleaned after brushing. An in vitro work identified the removal of 97% of the biofilm grown on titanium disks with modified surfaces, brushed with conventional toothbrushes [16]. Another in vivo study identified an average removal of 72.8% of biofilm in titanium healers with previously modified surfaces. According to the authors, on surfaces with greater roughness, the average biofilm removal was 52.6% [17]. Some points may explain these differences. First, the aforementioned studies brushed less complex surfaces, such as titanium discs and implant abutments. Second, brushing was performed with toothbrush and toothpaste. Furthermore, the brushing technique applied by Park et al. (2013) consisted of performing 40 unilateral strokes [16]. Elter et al. (2008) did not standardize the brushing method [17]. In the present study, dentifrice was not used, so that the removal of the simulated biofilm resulted exclusively from the mechanical action of the bristles. The brushing technique employed consisted of fewer movements, and, in the proximal sites only horizontal movements were performed. Finally, the hemi‐arches used in this study presented a more complex condition to be cleaned, which may have hindered the action of the bristles.

The influence of implantoplasty on simulated biofilm removal did not occur uniformly between the cervical, middle and apical thirds. The cervical thirds of the buccal/oral sites were the only ones with statistically significant reduction. Even so, the other thirds of these sites also showed reduction, but without statistical significance. This fact can be explained by the topography of the implant after implantoplasty. The convex burs resulted in a portion of the middle and apical thirds of the selected area being concave, which may have reduced the action of the bristles given that less pressure would have been applied to those areas.

According to an intragroup analysis of the cervical, middle and apical thirds of the proximal sites, greater biofilm removal was identified in the test group in the middle and apical thirds when compared to the cervical third (*p < 0.001*). In the control group, the apical third showed greater reduction of simulated biofilm when compared to the other thirds of the same face (*p < 0.009*). The less favourable results found on the proximal sites may be related to the brushing technique applied on these surfaces, which consisted of fewer horizontal strokes. On the other hand, a research carried out by Kim et al. (2022) identified a limit in the amount of simulated biofilm removal on the proximal surfaces of acrylic teeth, after a certain number of repetitions performed with an interdental brush [25]. According to the aforementioned research, there was a significant difference in the amount of simulated biofilm removed with only up to 5 back‐and‐forth strokes. A higher number of repetitions did not result in a significant reduction of biofilm on these sites [25].

After implantoplasty, the proximal space became obviously larger. For standardization, the same diameter of interdental brushes was used for both the test and control groups. It might be possible that the worst results of proximal sites are associated with the diameter of the interdental brushes, although the interdental brushes utilized fit the proximal areas of both groups. Further studies should address larger diameters for proximal brushing of implants submitted to implantoplasty.

A possible negative influence on the removal of biofilm from the cervical third of the proximal sites could be the angle of the emergence profile between the implant and the provisional crown. One study identified that angles between 30° and 50° resulted in a greater amount of biofilm, even under regular cleaning [26]. For standardization purposes, the present study used the same crown in all tested implants, which may justify the reproduction of this pattern in all specimens. Future studies with crowns with different angles may give a different perspective for the subject studied.

Another pattern observed on the buccal/oral and proximal sites of the implants in the control group was the disposition of the remaining simulated biofilm. The images suggest that peaks of the threads were cleaned better than the valleys. However, this cannot be stated since the applied methodology did not allow for the limits of peaks and valleys to be accurately identified. Future studies should be made in order to topographically analyse the residual biofilm after brushing intact implants.

In vitro research allows for the analysis of conditions that are difficult to reproduce clinically. On the other hand, this research had certain limitations. The simulated biofilm used in this study cannot be compared with in vivo biofilm in terms of adhesion to the implant surface. However, this is an appropriate way to simulate biofilm in an in vitro study, as was done in other studies. Moreover, the surface analysis of the implants after implantoplasty was performed under visual magnification rather than a quantitative method. An analysis of surface roughness would provide additional information to the study. However, after implantoplasty and polishing, the implant surface was considerably smoother than in the control group, and this is how it would appear clinically. The brushing of the specimens was performed by an experienced professional in a controlled environment; only one brushing method was investigated, so it is not possible to infer that other methods, or time spent on brushing, would reproduce the same results. The operator's position at the time of brushing did not reproduce the position of an individual brushing their own teeth; however, it reproduced the position of a caregiver cleaning an individual's mouth. Additionally, the model used in the study did not provide soft tissues, like lips, mucosa and tongue, that might impair the efficacy of brushing in a clinical situation. The photographs of the implants were taken from a single angle, which may have influenced the accuracy of the biofilm analysis in the control group, but without relevance in the test group. Although these conditions can be considered limitations of this study, they also help to control variations that are present in clinical studies. That said, it is recommended that the results obtained be considered with caution, and that clinical studies be carried out in the future, according to the above‐mentioned recommendation, to confirm the results found in this study.

## 5 Conclusion

It can be concluded that implantoplasty positively influenced the removal of simulated biofilm on the buccal/oral sites of dental implants.

## 6 Clinical Relevance

### 6.1 Scientific Rationale for the Study

It is known that biofilm plays a crucial role in dental implant longevity. The intraosseous portion of the implant that is exposed to the oral cavity, mainly because of peri‐implant disease, became a potential factor for biofilm accumulation and disease progression. However, there are few studies that investigate the cleaning efficacy of these surfaces.

### 6.2 Principal Findings

The results showed that implantoplasty favoured the cleaning mainly on buccal/oral sites.

### 6.3 Clinical Implications

Implantoplasty demonstrated to be a favourable alternative to facilitate home care cleaning of exposed surfaces of implants. However, clinical studies are needed to confirm these findings.

## Author Contributions

R.I.R.: concept/design, data collection, data analysis/interpretation, statistics and drafting article. C.O.S.: data interpretation, drafting article, critical revision of article, approval of article.

## Funding

This study was financially supported by Curaden/Curaprox, which provided toothbrushes and interdental brushes as well. By S.I.N. Implant System, which provided the dental implants. By Microdont, which provided the burs. By Bausch BK, which provided the occlusion spray.

## Conflicts of Interest

The authors declare no conflicts of interest.

## Data Availability

The data that support the findings of this study are available on request from the corresponding author. The data are not publicly available due to privacy or ethical restrictions.
